# Role of diode laser (810 nm) and/or fluoride varnish for the treatment of gamma-irradiated hypersensitive human dentin (An in vitro study)

**DOI:** 10.1038/s41598-025-10493-1

**Published:** 2025-07-10

**Authors:** Hoda Nabil Ahmed, Ahmed Abbas Zaky, Lamia E. Daifalla, Mostafa A. Bakr

**Affiliations:** 1 Al Zahraa Specialized Dental Center, Egyptian Ministry of Health and Populations, Cairo, Egypt; 2 Medical Applications of Laser Dept, National Institute of Laser Enhanced Sciences (NILES), Cairo University, Giza, Egypt; 3 Conservative Dentistry Dept, Faculty of Dentistry, Helwan National University, Cairo, Egypt; 4 Health Radiation Research Dept, National Center for Radiation Research and Technology (NCRRT), Egyptian Atomic Energy Authority (EAEA), Cairo, Egypt

**Keywords:** Diode laser, Fluoride varnish, Gamma radiation, Dentin hypersensitivity, Environmental scanning electron microscope, Health care, Medical research

## Abstract

Head and neck cancer is the sixth most frequent cancer worldwide. Dentin hypersensitivity (DH) is a common complication in patients undergoing radiotherapy. Gamma radiation significantly affects dental hard tissues, particularly dentin. Management of DH can be achieved using different agents such as fluoride and laser. This study aimed to evaluate the effectiveness of diode laser (810 nm), fluoride varnish, and their combination in managing DH in gamma-irradiated hypersensitive human dentin. Forty dentin specimens were assigned into four groups, G; gamma-irradiation only, GF; gamma-irradiation then fluoride varnish, GL; gamma-irradiation then laser, GFL; gamma-irradiation then fluoride then laser. Specimens were qualitatively assessed before and after citric acid challenge using environmental scanning electron microscope (ESEM). Image analysis for ESEM photomicrographs was performed to quantitatively evaluate the percentage of open dentinal tubules (DTs). The percentage of open DTs of group G before citric acid challenge showed the highest value that insignificantly increased after acid challenge. The percentage of open DTs of GF and GL groups significantly increased after citric acid challenge. The percentage of open DTs of group GFL insignificantly increased after citric acid challenge. Groups can be arranged according to the percentage of open DTs descendingly before citric acid challenge: G > GL > GFL > GF and after citric acid challenge: G > GF > GL > GFL. The combination of diode laser and fluoride varnish proves most effective in maintaining DTs occlusion following citric acid, offering a promising strategy for managing DH in patients undergoing radiotherapy.

## Background

Head and neck carcinomas (HNC), especially those of the oral cavity, are moderately prevalent and rising globally. Head and neck squamous cell carcinoma (HNSCC), the most common subtype, is the sixth most frequent cancer worldwide, causing around 890,000 new cases and 450,000 deaths annually^1^. Its incidence is expected to rise by 30% annually by 2030, with oral cavity cancers among the top three cancers in high-burden regions^2^. In Egypt, HNSCC shows distinct patterns linked to demographic and healthcare factors. Data from the national cancer registry (1999–2006) showed a higher incidence of HNSCC in urban areas, with males being more affected overall, while females are more prone to developing gum and mouth cancers^3^.

Owing to the increasing number of HNC patients, besides the higher cure rates after radiotherapy (RT), it is essential to realize the effect of RT on oral tissues. Although RT has an important role in managing HNC, it has many devastating dental-oral side effects. Radiotherapeutic patients are susceptible to hyposalivation, oral mucositis, loss of taste, dental caries, trismus, and osteoradionecrosis, as well as the development of radiation-associated dentin hypersensitivity (DH)^4^. Furthermore, RT directly and indirectly affects dentin, leading to DH. Direct effects include decreased microhardness, alteration in collagen structure, and obliteration of dentinal tubules (DTs). Indirect effects include reduced salivary flow, altered oral microbiota, and compromised pulp vitality, all contributing to increased dentin exposure and sensitivity^5^.

DH remains a challenging condition to manage, especially in patients undergoing RT for HNC, where dentin’s microstructure and organic content are significantly altered^6^. Recent advances in biomaterials have opened new avenues for treatment, offering enhanced remineralization and tubule occlusion capabilities. Among these, bioactive glasses modified with adhesives or gelatin matrices have the potential to form hydroxyapatite layers similar to natural dentin^7,8^. Combining bioactive glass with laser therapies (e.g., Nd: YAG or CO₂ lasers) has synergistic effects in sealing DTs and protecting the collagen matrix^9^. In addition, novel nanotechnologies, such as photothermal conversion nanoparticles and strontium-substituted calcium phosphate spheres, have emerged as effective agents in reducing DH through deep DTs penetration and apatite layer formation^10^.

Fluoride varnish is one of the most widely used topical desensitizing agents due to its ability to occlude DTs and reduce fluid movement inside them^11^. The application of fluoride varnish primarily acts by depositing calcium fluoride-like crystals on the dentin surface and within DTs, thereby physically blocking them and reducing fluid movement that triggers sensory responses. Additionally, fluoride enhances remineralization, restoring the mineral content compromised by irradiation^12^.

Compared to other means of controlling DH, the laser is characterized by being painless, easy, safe, and dependable, besides having an immediate effect^13^. Diode lasers (typically operating at wavelengths around 810–980 nm) exert a photothermal effect that promotes protein coagulation and fusion of peritubular dentin, effectively sealing DTs^13,14^.

The synergistic use of diode laser and fluoride varnish may offer a dual action approach owing to the immediate DTs occlusion by laser-induced coagulation and sustained desensitization through fluoride-mediated remineralization^13,14^. To date, several studies have investigated the use of diode laser combined with fluoride in managing DH in non-gamma-irradiated teeth^15–17^; however, no studies specifically evaluated laser therapy alone or in combination with fluoride in treating hypersensitive gamma-irradiated human dentin.

In light of the identified research gaps, the null hypotheses of the present study are as follows: (1) the combined treatment approach, including both 810 nm diode laser and fluoride varnish, will not yield any curing effect on gamma-irradiated hypersensitive dentin, (2) the citric acid challenge will not affect the percentage of open dentinal tubules. Thus, this study aimed to evaluate the effectiveness of diode laser, fluoride varnish, and their combination in managing dentin hypersensitivity in gamma-irradiated hypersensitive human dentin, filling a gap in the literature regarding treatment strategies for dentin hypersensitivity in radiotherapeutic patients.

## Methods

### Ethical considerations

The experiment was conducted in accordance with the protocol approved by the Ethical Committee of the National Institute of Laser Enhanced Sciences (NILES), Cairo University, Giza, Egypt, under approval reference NILES-EC-CU 24/6/14.

### Sample size calculation

According to the previous studies^18,19^, minimum total sample size of 40 samples is sufficient to detect the effect size of 0.33, with a power (1-β = 0.90) at a significance probability level of *p* ≤ 0.05 to evaluate the effectiveness of diode laser, fluoride varnish, and their combination in managing dentin hypersensitivity in gamma-irradiated human dentin. According to sample size calculations, there is a 90% chance of correctly rejecting the null hypothesis of no significant effect if each group is represented by 10 samples. The sample size was calculated according to G*Power software version 3.1.9.7.

### Teeth selection and grouping

Forty dentin specimens were prepared from twenty sound human premolar teeth extracted for periodontal reasons following the acquisition of informed consent from the donors. Teeth were examined visually and by transillumination to ensure they were free from decay, enamel malformations, cracks, decalcification, or restoration. 2.5 mm of the buccal, lingual, and occlusal enamel of teeth was removed and verified by a digital caliper (Mitutoyo, Tokyo, Japan) till reaching the dentin layer to simulate exposed dentin, utilizing a precision saw under copious air/water cooling (Isomet^®^4000 Linear Precision Saw, Buehler 11-2680 Ltd., Lake Bluff, IL, USA). To achieve tooth surface flattening, sequential grinding was performed using 320, 600, 1000, and 1200 grit sandpapers (Sia Abrasives Industries, Switzerland) in ascending order of fineness. Smoothening was manually performed using a polishing paste (Alpha-Pro prophylaxis paste, USA) and a wet 1200-grit sandpaper^18^.

Afterward, teeth were vertically sectioned mesiodistally, then horizontally trimmed over the enamel cementum junction so that each tooth ended up into two dentin specimens^18^. Specimens were etched using 37% phosphoric acid etching gel (META Biomed Co., Ltd., Korea) for 20 s to fully open the dentinal tubules (DTs) and remove organic materials^20^. The application of 20–40% phosphoric acid to dentin exposes a demineralized collagen network of 6–9 μm thick in dentin^21^. Following etching, specimens were rinsed with distilled water for 1 min^20^.

Specimens were randomly and equally assigned into four groups (*n* = 10): G group, specimens were exposed to gamma-irradiation only; GF group, specimens were exposed to gamma-irradiation then fluoride varnish; GL group; specimens were exposed to gamma then laser irradiation; GFL group, specimens were exposed to gamma-irradiation then fluoride varnish followed by laser irradiation. Dentin specimens were examined after treatment with 37% phosphoric acid etching gel followed by the applied treatment. Each group underwent micromorphological evaluation at two stages: following the respective treatment (before the citric acid challenge) and after exposure to the citric acid challenge.

### Gamma-irradiation

Specimens were subjected to gamma-irradiation at a total dose of 60 Gy, which is the therapeutic dose for head and neck cancer patients^22^. Gamma-irradiation was performed at the National Centre for Radiation Research and Technology (NCRRT), Egyptian Atomic Energy Authority, Cairo, Egypt, using the ^60^Co Gamma Cell (220, India) at a dose rate of 0.657 KGy/h at the time of the study.

### Fluoride varnish application

A 5% sodium fluoride varnish (Charm Varnish^®^ – Dentkist, Korea) was applied to the surfaces of all specimens in the GF and GFL groups using standardized micro-brushes. The varnish was left on the specimen surfaces for five minutes before the excess was removed. Immediately afterward, the specimens in the GFL group were subjected to laser irradiation^23^.

### Laser irradiation

Twenty-four hours after gamma-irradiation, laser irradiation of the specimens was performed at the National Institute of Laser Enhanced Sciences (NILES), Giza, Egypt. Specimens of groups GL and GFL were exposed to diode laser (Zolar 810 nm, Photon series, Zolar Technology & Mfg Co. Inc., Canada) at one-watt power for 30 s, continuous wave (CW) mode using an optic fiber transmission system (400 nm) in the non-contact mode. The tip was positioned perpendicular to the surface of each specimen and then longitudinally and uniformly moved in a scanning motion over the entire surface^24^.

### Citric acid challenge

Following treatment, all specimens were exposed to the citric acid (6% wt.) challenge for one minute under dynamic conditions. The citric acid was freshly prepared of six g citric acid monohydrate powder (El Nasr Pharmaceutical Chemicals Co., Egypt) added to 100 mL of deionized water (pH was adjusted at 4)^20^. The citric acid solution was used to check the resistance of the applied treatments to the strongly acidic environment^25^. Next, specimens were rinsed using distilled water and left to air dry for 24 h before being subjected to environmental scanning electron microscope (ESEM) assessment^23^.

### Study assessments

Each dentin specimen was assessed before and after the citric acid challenge. ESEM was combined with digital image analysis to provide qualitative and quantitative assessment. ESEM (Prisma E, Thermo Fisher Scientific Inc., USA) attached to the EDX unit to assess each dentin specimen’s surface morphology. Specimens were fixed on aluminum stubs with standard diameter using a carbon double sticky tape. ESEM examination of each specimen was operated at an accelerating voltage of 30 kV. The examination of all groups was performed at x1000 magnification. Representative photomicrographs of all groups under standardized working distance were selected. Image analysis for ESEM photomicrographs was processed using Image J software (version 1.53a National Institutes of Health, USA) to quantitatively calculate the open DTs percentage. The total image area was measured in µm^2^, and then the total area of opened DTs was calculated as a percentage of the total image area using the following equation:

$$\:\text{O}\text{p}\text{e}\text{n}\:\text{d}\text{e}\text{n}\text{t}\text{i}\text{n}\text{a}\text{l}\:\text{t}\text{u}\text{b}\text{u}\text{l}\text{e}\text{s}\:\text{\%}=\frac{Total\:area\:of\:open\:dentinal\:tubules\:\:\left(\mu\:m2\right)}{Total\:image\:area\:\left(\mu\:m2\right)}\times100\:$$

### Statistical analysis

To evaluate the effectiveness of diode laser, fluoride varnish, and their combination in managing dentin hypersensitivity in gamma-irradiated hypersensitive dentin in four main groups (G, GF, GL, and GFL) at two measurement points (Before and after the citric acid challenge), image analysis results were statistically analyzed using one-way analysis of variance (ANOVA) to indicate intra-group significance, two-way repeated measures ANOVA analysis to indicate the interaction between treatment type and time point (inter-group comparisons), followed by Tukey’s multiple comparisons post hoc test to indicate inter-group significance. *p* ≤ 0.05 was considered statistically significant (95% significance level), and *p* ≤ 0.001 was considered highly statistically significant (99% significance level). Statistical evaluation was performed using the SPSS statistical package (version 25, IBM Co. USA).

## Results

The substrates under investigation were hypersensitive gamma-irradiated human dentin specimens. They were assigned into four groups according to the applied treatment. Specimens were examined for open dentinal tubules (DTs) using an environmental scanning electron microscope (ESEM) twice, after each treatment (before the citric acid challenge), and after the citric acid challenge for qualitative and quantitative assessments.

### Effect of different treatments on dentinal tubules patency

**Fig. 1:**
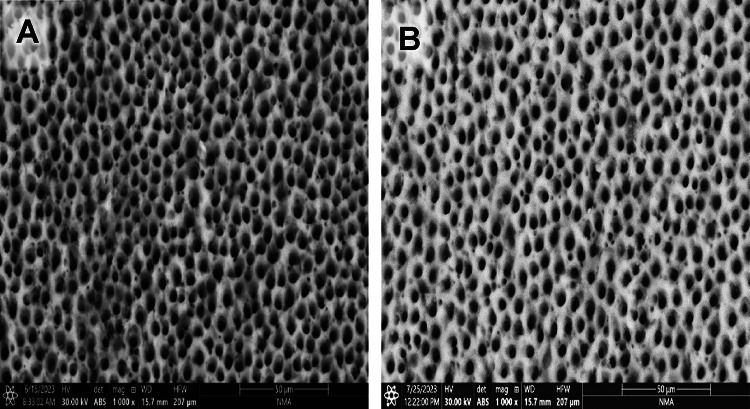
Scanning electron micrograph of group G showing open DTs before and after the citric acid challenge (A, B) (x1000).

**Fig. 2:**
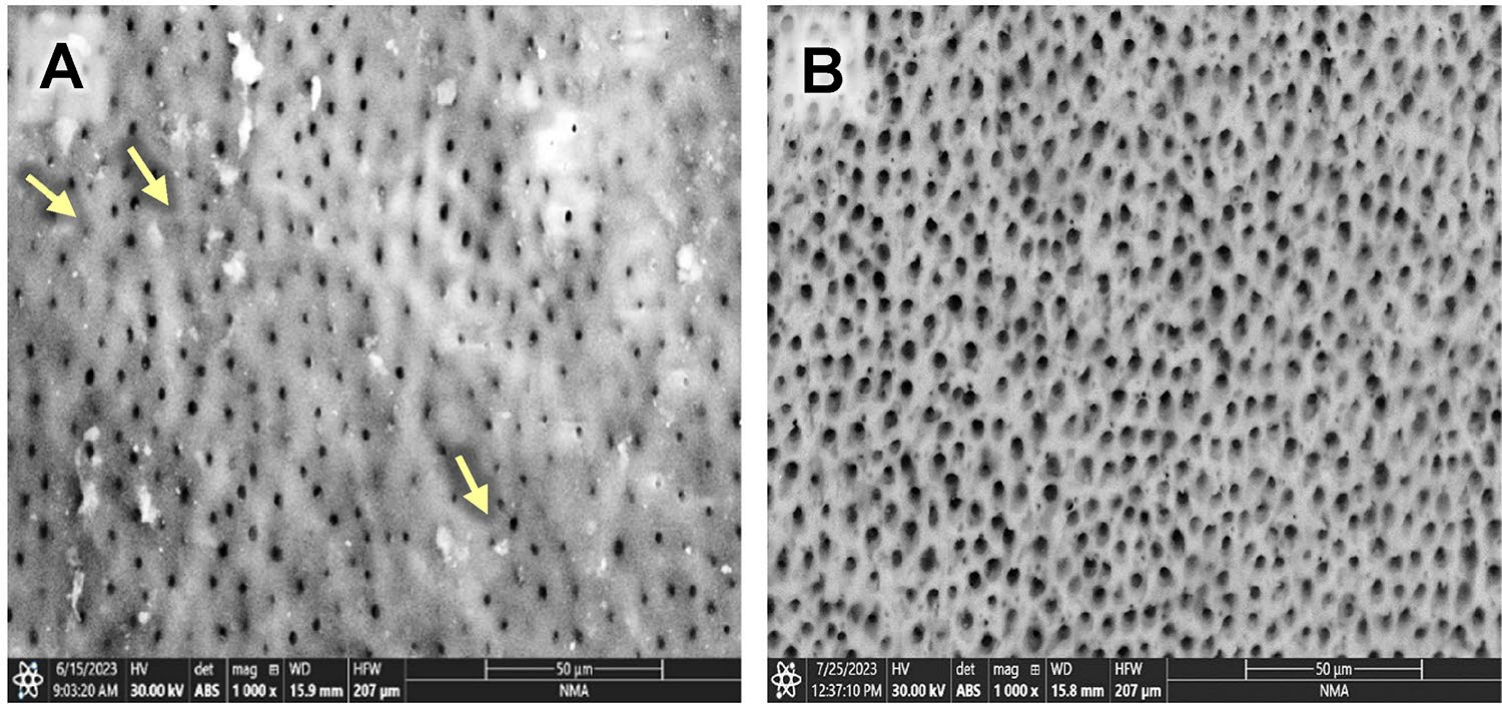
Scanning electron micrograph of group GF showing obliterated DTs in some areas (yellow arrows) and opened ones in others before the citric acid challenge (**A**). In contrast, after the citric acid challenge more opened DTs were displayed than before the citric acid challenge (**B**). (x1000).

**Fig. 3:**
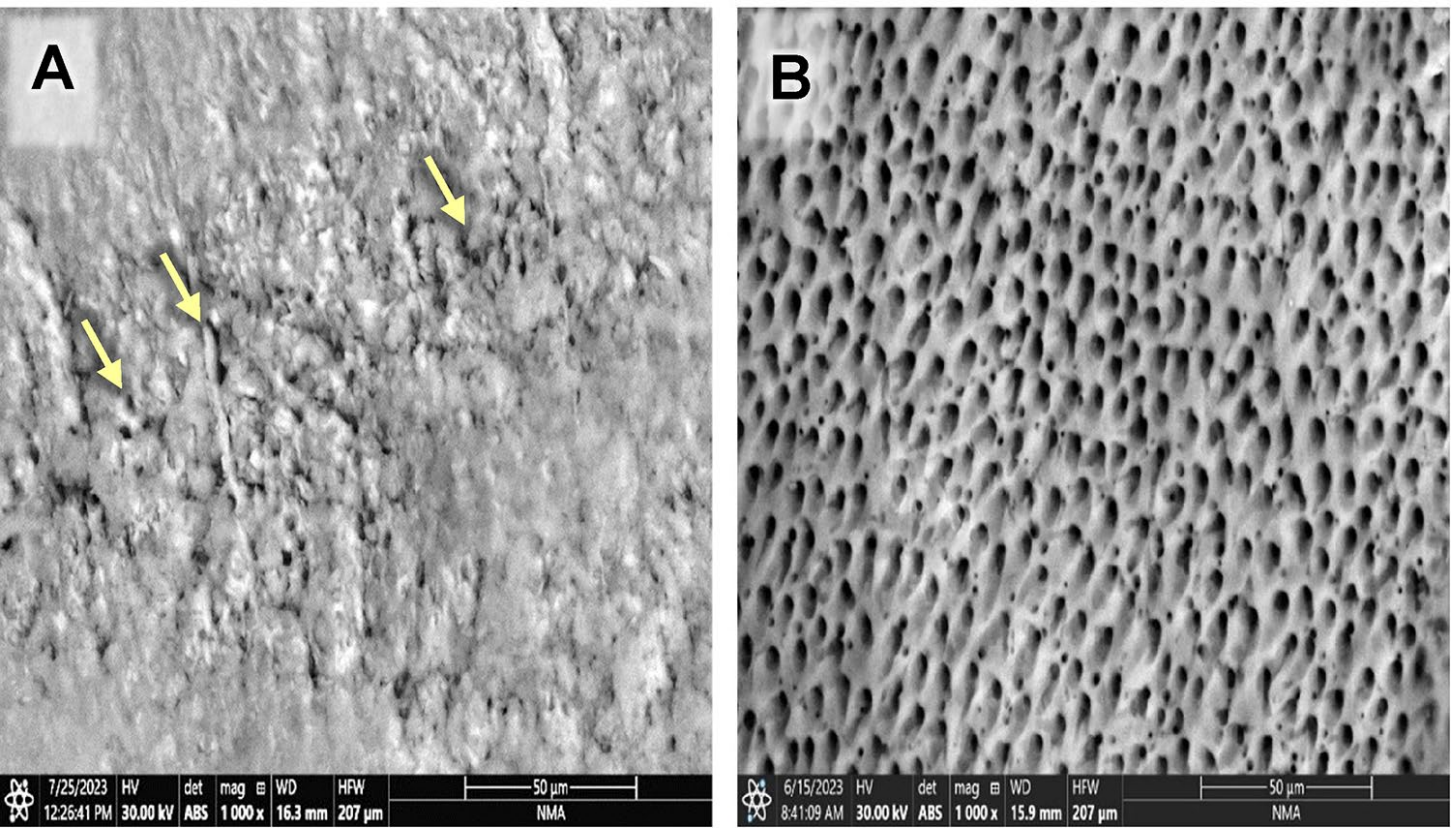
Scanning electron micrograph of group GL showing obliterated DTs in some areas while others remained open before the citric acid challenge (yellow arrows) (**A**), and open DTs with irregular ends after the citric acid challenge (**B**) (x1000).

**Fig. 4:**
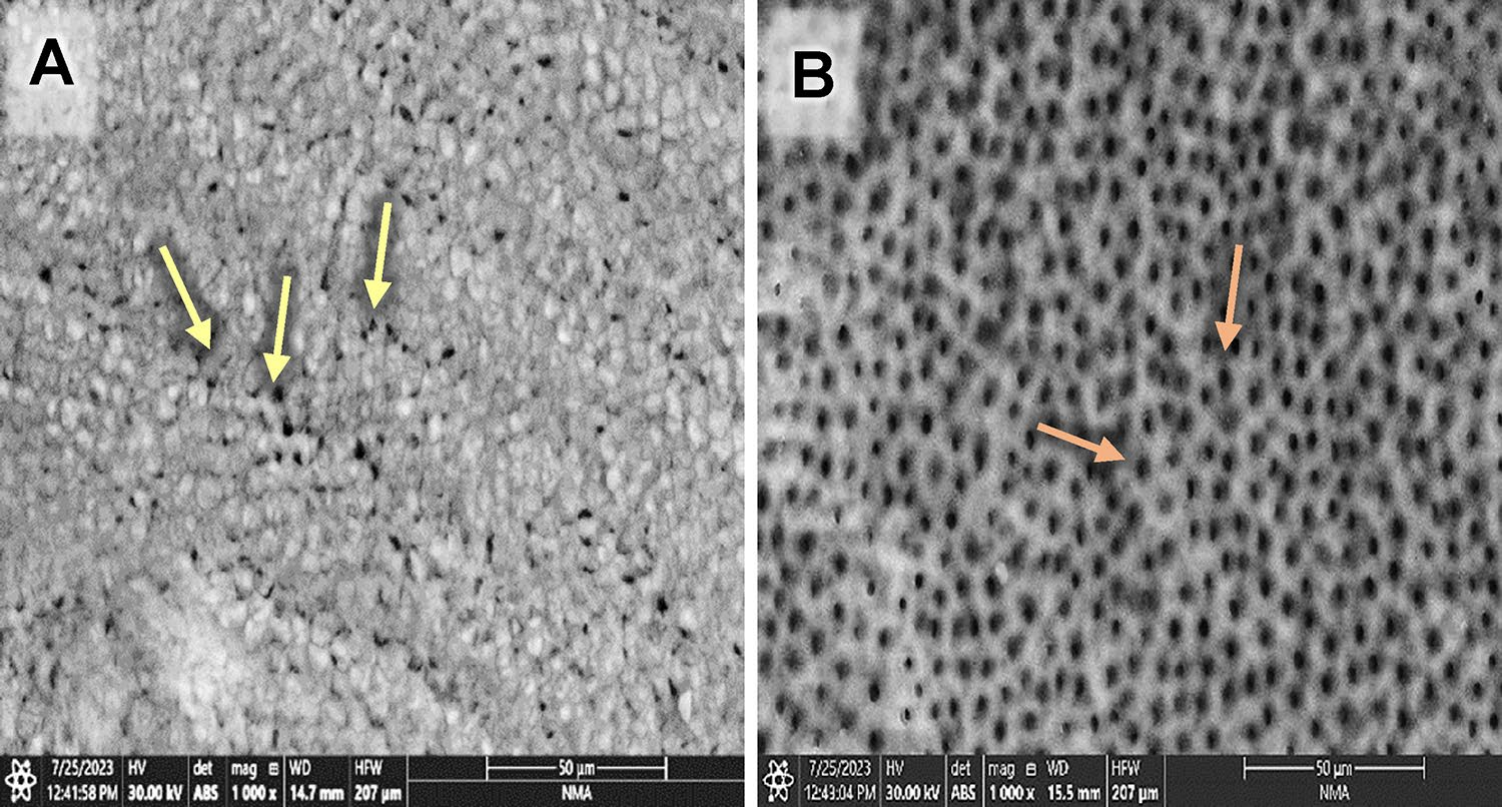
Scanning electron micrograph of group GFL showing open DTs in some areas (yellow arrows) and obliterated ones in others before citric acid challenge (**A**), and open DTs in some areas (red arrows) and obliterated ones in others after the citric acid challenge (**B**) (x1000).

Evaluating the photomicrographs of dentin specimens by ESEM revealed obvious open DTs in group G before and after the citric acid challenge (Fig. 1A& B). However, regarding GF group, several obliterated DTs (yellow arrows) were shown in some areas, added to some opened DTs in others before the citric acid challenge. In contrast, after the citric acid challenge, more opened DTs were displayed (Fig. 2A& B). Concerning group GL, obliterated DTs were noticed in some areas while others remained open before the citric acid challenge (yellow arrows); also, open DTs with irregular ends were observed after the citric acid challenge (Fig. 3A& B). On the other hand, GFL showed open DTs in some areas (yellow arrows) and obliterated ones in others before the citric acid challenge. Similarly, after the citric acid challenge, open DTs in some areas (red arrows) and obliterated ones in others were detected (Fig. 4A& B).

### Effect of different treatments on the percentage of open dentinal tubules

**Fig. 5:**
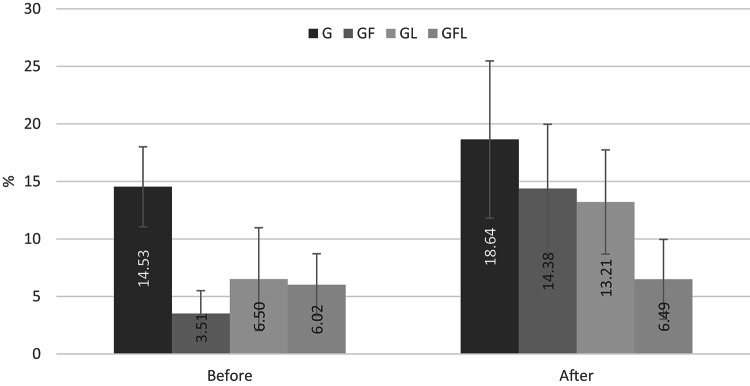
A bar chart representing the mean percentage of open dentinal tubules for all groups before and after the citric acid challenge.

The quantitative analysis of open DTs before and after the citric acid challenge revealed statistically significant differences among the treatment groups. Before the acid challenge, group G exhibited the highest mean percentage of open DTs (14.47%), significantly greater than the GF (3.51%, *p* = 0.0018), GL (6.50%, *p* = 0.0292), and GFL (6.02%, *p* = 0.0192) groups. No significant differences were observed among the GF, GL, and GFL groups at this stage (*p* > 0.05), indicating similar effectiveness in tubule occlusion (Table 1 & Fig. 5).

After the citric acid challenge, GFL group demonstrated the lowest mean percentage of open DTs (6.49%), indicating the most significant resistance to the effect of the citric acid, compared to G group (17.85%, *p* = 0.0012), and GF group (14.38%, *p* = 0.0219). The GL group also showed a lower mean percentage of open DTs (13.21%) than G group, but the difference was not statistically significant (*p* = 0.3294). The difference between GF and GL was also insignificant (*p* > 0.05). However, the GL vs. GFL comparison approached significance (*p* = 0.0616) (Table 1 & Fig. 5).

### Effect of the citric acid challenge on the percentage of open dentinal tubules

Image analysis of the photomicrographs of dentin specimens related to group G showed the highest percentage of open DTs before the citric acid challenge that insignificantly increased after the citric acid challenge (*p* > 0.05). Regarding GF group, it showed a highly significant increase in the percentage of open DTs after the citric acid challenge (*p* = 0.001). Similarly, the percentage of open DTs in group GL significantly increased after the citric acid challenge (*p* < 0.05). However, the percentage of open DTs of GFL group insignificantly increased after the citric acid challenge (*p* > 0.05) (Table 1 & Fig. 5).

The mixed-effects model analysis confirmed that treatment type and time significantly affected the percentage of open DTs (*p* = 0.0167 and 0.0009, respectively). However, the interaction between treatment and time was not statistically significant (*p *= 0.0674). The predicted mean percentage of open DTs increased significantly from 7.62% before treatment to 12.98% after the acid challenge, with a mean difference of -5.36% (*p* < 0.05). These results suggest that while all interventions reduced the mean percentage of open DTs compared to the gamma-irradiation group, the combined fluoride varnish and diode laser treatment (GFL group) provided the most effective and acid-resistant DTs occlusion.

**Table 1:** Mean ±SD and intra-group comparison of the percentage of open dentinal tubules for all groups before and after the citric acid challenge.

Time	G	GF	GL	GFL
Before	14.47±3.48^a^	3.51±1.99^b^	6.5±4.47^b^	6.02±2.69^b^
After	17.85±6.83^a^	14.38±5.59^a^	13.21±4.53^ab^	6.49±3.47^b^
P-value*	0.406^NS^	0.001^HS^	0.027^S^	0.797^NS^

*Overall P-value for Intra-group comparison (ANOVA test).

NS= Non-significant at *P* < 0.05, S= Significant at *P* ≤ 0.05, HS= Highly significant at *P* ≤ 0.001.

In the same row, different letters mean significant.

## Discussion

The present study investigated the efficacy of diode laser, fluoride varnish, and their combination in managing dentin hypersensitivity (DH) in gamma-irradiated hypersensitive human dentin. According to our results, the first null hypothesis was rejected as the combined treatment approach, including 810 nm diode laser and fluoride, proves most effective in maintaining dentinal tubules (DTs) occlusion. Whereas the second hypothesis can be partially rejected as an increase in the percentage of open DTs following the citric acid challenge was detected.

Environmental scanning electron microscopy (ESEM) demonstrated distinct surface modifications across treated groups. In gamma-irradiated dentin, morphological degradation was evident as widened, irregular DTs. These changes are attributed to dehydration, collagen shrinkage, and mineral loss due to radiation exposure^5,26^. Fluoride varnish created crystalline deposits that partially occluded tubules, consistent with the precipitation of CaF₂-like particles^27^. Laser irradiation caused melting and solidification of peritubular dentin, leading to mechanical sealing of tubule orifices^28,29^.

Before the citric acid challenge, the results revealed a high percentage of DTs openings, considering the gamma-treated specimens compared to all other groups. Gamma radiation is known to induce substantial structural and compositional alterations in dental hard tissues. These alterations include degradation of collagen fibers, reduced water content, demineralization, and increased permeability due to widened DTs^30^. This result may be due to the degeneration of the organic contents that might disturb the link between enamel and the enamel dentin junction giving rise to the instability of the adjacent area and the impairment of its mechanical properties, and as a result, exfoliation of enamel and exposure of dentin come about^31^.

In contrast to our results, several authors have detected partial or total obliteration of DTs after 30 and 70 Gy gamma-irradiation^5,26,32^. The dissimilarity may be correlated to different anatomical regions for acquiring dentin specimens or different irradiation doses. Additionally, a previous study pointed out that no significant statistical difference was detected between teeth specimens subjected to 20 KGy gamma-irradiation (Co^60^) and control regarding the morphological changes of the DTs^33^. However, those morphological changes were visually examined, yet in our study, we used image analysis for detecting DTs morphological changes.

Nevertheless, no statistically significant differences were detected in DTs openings comparing GF, GL, or GFL groups (*p* > 0.05), before the citric acid challenge. This finding may be attributed to the biological changes and the structural damage induced by gamma radiation, which diminishes the dentin’s capacity to respond to the tested desensitizing agents. Moreover, these biological changes can compromise dentin’s natural defense mechanisms and limit the effectiveness of conventional desensitizing treatments, particularly those relying on physical occlusion or chemical bonding^34^.

At the same time, the mechanistic action of fluoride varnish and diode laser, either in combination or individually used, has some limitations. Fluoride varnish works primarily by forming calcium fluoride-like crystals that occlude DTs and promote remineralization^35^. However, in gamma-irradiated dentin, reduced mineral content and altered collagen may reduce fluoride uptake and retention. The varnish may not adhere well to denatured or highly porous dentin surfaces^36^. This limited adherence is likely a consequence of radiation-induced microstructural changes that hinder proper adhesion and infiltration.

Similarly, the diode laser works by inducing physical changes such as protein coagulation and partially melting the DTs surface, leading to their occlusion^37^. Nevertheless, these effects may be attenuated in irradiated dentin, where thermal conductivity and the chemical structure of dentin are already compromised^38^. The altered surface morphology and reduced moisture content may affect laser-tissue interaction and increase the risk of thermal damage or inconsistent treatment outcomes. Variability in laser parameters, including wavelength, power, and exposure time, further complicates reproducibility and long-term reliability in clinical scenarios^39^. Furthermore, the combination of fluoride varnish and diode laser, which is often thought to provide synergistic benefits through simultaneous chemical and physical tubule occlusion, may not yield added efficacy in irradiated dentin due to the altered substrate response.

In accordance with our results, a recent in vitro study proved that fluoride extensively coated occluded patent DTs and as a result, provided a significant reduction in DH after 8 weeks of use^40^. In the same way, significant DTs occlusion of dentin specimens treated with fluoride was verified and confirmed by SEM^41^. Fluoride ions (F–) are smaller than the diameter of DTs^42^. This may explain the lowest mean of open DTs obtained in GF group before the citric acid challenge. In addition, many previous studies suggested that 810 nm diode laser irradiation is promising in reducing the diameters of DTs and as a result, could be used for treating DH^28,43^.

After the citric acid challenge, the percentage of open DTs insignificantly increased in gamma-irradiated dentin. This could be attributed to increased brittleness, pre-existing cracks, and altered surface morphology, where the denatured collagen matrix and the compromised mineral content can limit acid penetration^30^. Bakr et al.^18^ demonstrated that the microhardness of gamma-irradiated human tooth structure significantly declined after exposure to acidic environment.

Evidence from the results showed that while fluoride varnish is effective in occluding DTs, its efficacy diminishes dramatically upon exposure to acidic environments. This led to a statistically significant increase in the percentage of open DTs, compromising the effectiveness of the desensitizing treatment. This effect is primarily due to the acid’s ability to dissolve or dislodge the mineral precipitates formed by fluoride varnish, leading to increased dentin permeability. Recent research comparing innovative desensitizing agents indicates that fluoride-based treatments are less resistant to acid challenges than other agents like bioactive glasses^44,45^. This suggests the vulnerability of fluoride-induced occlusions to acidic conditions.

Regarding 810 nm diode laser irradiation, the results of the present study showed that the percentage of DTs opening significantly increased in hypersensitive gamma-irradiated human dentin following exposure to citric acid challenge. This result can be explained through several mechanisms. Citric acid is a chelating agent used to remove the smear layer and expose DTs^46^. Besides, 810 nm diode lasers have been shown to thermally affect dentin by melting and fusing peritubular dentin, potentially occluding DTs when used alone. However, when applied after demineralization (as in citric acid conditioning), the laser may enhance the removal of organic remnants and expose or enlarge already opened DTs rather than occluding them. This may be due to the enhanced thermal interaction with dentin^37^. The diode laser can induce superficial melting and re-solidification of the dentin surface, leading to initial tubule sealing; this seal is generally less resistant to acidic environments compared to deeper modifications achieved by higher-energy lasers^47^.

On the other hand, the hydraulic conductance inside DTs had declined in laser irradiated groups after citric acid challenge. This was related to the reaction of citric acid with free calcium ions, giving rise to calcium citrate crystals that might interact with hydroxyapatite and cause DTs occlusion^48^. Therefore, the lack of statistical difference between the two treatment groups may be attributed to the similar vulnerability of both treatment modalities to acid-induced degradation.

Despite the results suggesting that all interventions reduced the mean percentage of open DTs compared to gamma-irradiation alone, the combined fluoride varnish and diode laser treatment (GFL group) provided the most effective and acid-resistant DTs occlusion. These findings align with the literature suggesting that diode laser and fluoride act synergistically for durable DTs occlusion. Our result mirrors Ipci et al.^49^ and Kawle et al.^50^, who concluded that the dual-treatment approach is better than laser or fluoride alone in longevity and effectiveness. A recent study supported our results where the control group exhibited more open DTs with higher diameters compared to the laser and fluoride-treated group using ESEM^24^. Also, a statistically significant increase in DTs occlusion of laser and fluoride combination group was revealed after examining the occlusion ability of 810 nm diode laser in combination with fluoride using ESEM^29^. Results also match observations by Bakr et al.^18^ and Siddiqui et al.^14^, where an improvement in microhardness and decrease in permeability was detected with dual treatments using laser and fluoride. In contrast, no significant difference was detected regarding the diameter of open DTs between 980 nm diode, Nd: YAG and Er: YAG lasers accompanied by fluoride compared to control^23^.

When diode laser and fluoride varnish were combined, the percentage of open DTs was reduced even after the citric acid challenge. This reduction might be related to the capability of the diode laser to enhance the penetration and fixation of fluoride ions into the dentin structure. Recent studies have explored the combined effects of laser therapy and fluoride varnish on gamma-irradiated human tooth structure. A 2022 study assessed the impact of diode laser therapy combined with fluoride varnish on gamma-irradiated primary teeth. Results indicated that this combination significantly improved microhardness and microstructural integrity compared to untreated irradiated enamel^51^. Combining diode laser (940 nm) with nanohydroxyapatite may offer an effective treatment for DH, owing to the strong tubular occlusion capabilities and stability in acidic laser conditions^37^.

Following the citric acid challenge, the percentage of open DTs in hypersensitive gamma-irradiated human dentin treated with 810 nm diode laser and fluoride showed insignificant improvement compared to those treated with 810 nm diode laser alone. This observation suggests that the additive effect of fluoride varnish enhanced the acid resistance of laser-induced DTs occlusion in irradiated dentin. The non-significant increase in open DTs after the citric acid challenge suggests that the occluding layer formed by the combined treatment is more acid-resistant. This could be due to the physical sealing from laser energy combined with chemically stable fluoride complexes, resulting in greater resistance to demineralization.

While the study contributes important preliminary data on managing DH in radiotherapeutic patients using diode laser and fluoride varnish, its in vitro nature does not replicate the complex biological environment of the oral cavity. Long-term evaluation is also needed to assess the stability of DTs occlusion over weeks or months.

Future research on fluoride varnish and diode laser in managing DH in gamma-irradiated human dentin should focus on long-term durability assessment and comparative efficacy with alternative desensitizing agents. Also, in vivo trials is important to simulate the complex biological environment of the oral cavity. Finally, establishing a standardized protocol for managing DH in radiotherapeutic patients, involving multidisciplinary collaboration between dental researchers, oncologists, and materials scientists is necessary to improve patient outcomes.

## Conclusions

Under the limitations of this study, it could be concluded that without an acidic attack, the fluoride varnish did not offer an extra occluding effect than the (810 nm) diode laser either used separately or in combination for hypersensitive gamma-irradiated human dentin. Meanwhile, the combination of diode laser and fluoride varnish proves most effective in maintaining dentinal tubules occlusion following the citric acid challenge, offering a promising strategy for managing dentin hypersensitivity in patients undergoing radiotherapy.

## Data Availability

All data generated or analyzed during this study are included in this published article.
